# A Multisite Randomized Controlled Trial of Mindfulness‐Based Stress Reduction in the Treatment of Posttraumatic Stress Disorder

**DOI:** 10.1176/appi.prcp.20180002

**Published:** 2018-09-13

**Authors:** Lori L. Davis, Charles Whetsell, Mark B. Hamner, James Carmody, Barbara O. Rothbaum, Rebecca S. Allen, Al Bartolucci, Steven M. Southwick, J. Douglas Bremner

**Affiliations:** ^1^ Research and Development Service Veterans Affairs Medical Center Tuscaloosa AL; ^2^ Department of Psychiatry and Behavioral Sciences University of Alabama School of Medicine Birmingham; ^3^ Birmingham; ^4^ Mental Health Service Ralph H. Johnson VA Medical Center Charleston SC; ^5^ Department of Psychiatry Medical University of South Carolina Charleston; ^6^ Department of Medicine Division of Preventive and Behavioral Medicine University of Massachusetts Medical School Worcester; ^7^ Department of Psychiatry and Behavioral Sciences Emory University Atlanta; ^8^ Alabama Research Institute on Aging and Department of Psychology University of Alabama Tuscaloosa; ^9^ Department of Biostatistics University of Alabama at Birmingham; ^10^ Veterans Affairs National Center for PTSD Connecticut VA Healthcare System West Haven; ^11^ Department of Psychiatry Yale University New Haven CT; ^12^ Mental Health Service Atlanta VA Medical Center Decatur GA

**Keywords:** Trauma‐ and Stressor‐Related Disorders, Posttraumatic Stress Disorder, mindfulness‐based stress reduction, complementary integrative treatment, meditation, present‐centered group therapy, Veterans

## Abstract

**Objective:**

Posttraumatic stress disorder (PTSD) is often difficult to treat, and many patients do not achieve full remission. Complementary and integrative health approaches, such as mindfulness meditation, are intended to be integrated with evidence‐based treatment. This study examined the efficacy of mindfulness‐based stress reduction (MBSR) in the treatment of PTSD in U.S. military veterans.

**Methods:**

Veterans with a diagnosis of PTSD (N=214) were randomly assigned to either 90‐minute group MBSR or present‐centered group therapy (PCGT) for eight weeks. Follow‐up assessments were obtained at baseline and weeks 3, 6, 9 (primary endpoint), and 16.

**Results:**

Both the MBSR and PCGT groups achieved significant improvement in PTSD as measured by the Clinician‐Administered PTSD Scale for DSM‐IV (CAPS‐IV), with no statistically significant differences between groups. However, compared with PCGT, the MBSR group showed a statistically significant improvement in PTSD on the self‐reported PTSD Checklist for DSM‐IV over the nine weeks. This difference was not maintained posttreatment, at week 16. Strengths of the study include its large sample size, multisite design, active control group, single‐blind outcome ratings, fidelity monitoring, large minority representation, and randomized approach. The study was limited by its high attrition rate and low representation of women.

**Conclusions:**

Both MBSR and PCGT appear to have beneficial effects in treating PTSD in veterans, with greater improvement observed in self‐reported PTSD symptoms in the MBSR group. No differences between groups were observed on the CAPS‐IV scale.

Posttraumatic stress disorder (PTSD) involves an overgeneralization of a conditioned fight‐or‐flight response to previously neutral stimuli. Fear conditioning involves a neuronal circuit that once triggered, fires repeatedly, despite efforts to turn it off. This circuit becomes not only the path of least resistance, but also the final common pathway, firing automatically in response to stimuli that would be better served by a more flexible response. Psychotherapy uses the neuroplastic capabilities of the nervous system to facilitate the formation and strengthening of new neuronal circuits while weakening the connections among overlearned troublesome circuits (1, 2). In the case of PTSD, the firing of the overlearned circuit is accompanied by cognitive patterns that mark the disorder, such as believing the present situation to be unsafe, regardless of the circumstances. The habitual patterns of memory include cognitive representations of the trauma and accompanying strong emotions embedded in the limbic component of the circuit, leading to rage and fear responses (3).

Current treatments for PTSD include trauma‐focused psychotherapy and antidepressant medications, although the rates of response and remission to these interventions are mixed (4, 5). Approximately one‐third of PTSD patients discontinue medication or therapy prematurely because of difficulty tolerating the treatment. Many individuals living with a PTSD diagnosis are looking for alternatives to medication and/or trauma‐focused psychotherapy. Complementary and integrative health approaches, such as mindfulness meditation, have grown in popularity as low‐risk interventions used with evidence‐based medical treatments (6). Mindfulness‐based stress reduction (MBSR) is a technique taught in a series of classes that trains individuals to focus attention on thoughts, sensations, and feelings as they appear (7).

Incorporated into MBSR exercises such as body scan, sitting meditation, mindful yoga, and use of mindfulness in everyday life, mindfulness involves the intentional awareness of, and nonreactivity to, thoughts, sensations, and feelings as they arise. The focus of attention can be maintained on these mental/sensory contents and concomitant emotional responses, or the focus may be deliberately redirected to a different emerging thought, sensation, or feeling or to a wider field of awareness. This self‐regulatory behavior represents an openness to and acceptance of mental and sensory experiences that can change one's relationship with one's experience. Rather than remaining preoccupied with the content of mental or sensory experiences, one recognizes that events occurring in the field of awareness will, by their nature, change. For example, if a distressing memory is noticed, no attempt is made to change or suppress it; instead, these thoughts or feelings are noticed as one part of a broader range of experience in that moment. Thus, the attention required to sustain the thought and its attendant distress is re‐directed, preventing the escalation of negative thoughts into ruminative patterns. Mental space is left for more creative and less habitual or conditioned responses, which may contribute to a greater sense of control in stressful situations (8, 9, 10, 11, 12).

What is the rationale for using MBSR in the treatment of PTSD and by what mechanism could MBSR intervene in altering the habitual responses that comprise PTSD? The therapeutic process in the treatment of PTSD depends on the simultaneous presence of vivid experience and nonreactivity. Simple distraction from the distressing cognitions and emotions provides only temporary relief, as the underlying circuits remain intact and ready to fire whenever triggered. As with exposure therapy, in therapy for PTSD, it is essential that the trauma be actively brought to mind at the time of the treatment; however, trauma representation should be modulated by cognitive processes to avoid triggering the full trauma response, which would only reinforce the fear circuit. In the case of MBSR, traumatic memories that arise may be experienced within an accepting frame of mind and be less likely to induce the fear‐conditioned stress response. It is possible to experience aspects of traumatic recall, dysfunctional emotions, and sustaining cognitions without judgments such as “I’ll never be free of this.” By fostering an acceptance of raw experience, without trying to change it, MBSR decouples the experience from neuronal firings that code for defensive maneuvers and judgments about the experience (13). This acceptance allows the patient to be less reactive to the symptoms of PTSD (14). The unfinished business of the trauma is less likely to preoccupy the thought stream of the patient, and he or she is then free to pursue new experiences in life. Mindfulness meditation assists in decreasing rumination. As such, mindfulness may enhance self‐regulation and decrease emotional reactivity (15).

What is the benefit of a nontrauma‐focused intervention, such as MBSR? As pointed out by Brewer et al. (16), mindfulness training targets one's relationship with thoughts and the *process* of thoughts and feelings arising, whereas cognitive‐behavioral therapy intends to change the *content* of thoughts. Mindfulness incorporates cognitive reappraisal, which is an important component of cognitive‐behavioral treatments. In this case, the cognitive reappraisal involves learning to judge a traumatic memory as neither bad nor good. In addition, social support is known to buffer against symptoms of PTSD. Many mindfulness experts recommend forming a group practice to experience the full benefit of mindfulness. Group social support enhances one's sense of coherence (17) and facilitates compassionate behavior (18). Recent work has suggested that feelings of empathy for social pain are associated with increased activation on fMRI of the anterior insula and anterior cingulate cortex (19), regions of interest in the pathophysiology of PTSD.

Another key component of PTSD is avoidance. In many ways, mindfulness is the opposite of avoidance. Mindfulness meditation resembles an exposure situation in that “practitioners turn toward their emotional experience, bring acceptance to bodily and affective responses and refrain from engaging in internal reactivity toward emotional experience” (20). The MBSR practitioner does not try to avoid or push away whatever comes to mind but rather observes and focuses on mental images without judgment. MBSR uses one of the most important components of evidence‐based treatment for PTSD (i.e., exposure). However, MBSR does not intentionally or actively elicit traumatic memories, which may make it more palatable. Cognitive therapies are directive and ask that attention be given to challenging or even aversive material within a timeframe dictated by the constraints of a scheduled psychotherapy session. Such direction of attention may not be in accord with a patient's willingness, interest, or capacity to engage in difficult material. If there is a mismatch between the timing of the therapeutic intervention and the patient's readiness to process traumatic events, resistance arises (21). Cognitive therapy, therefore, is more likely to elicit greater resistance than mindfulness, which is inherently self‐directed. Although mindfulness practice is not trauma focused, it is trauma inclusive. Unpleasant experiences that need resolution arise naturally during the practice, as the patient's defenses relax. This relaxation of defenses is generally coordinated with a readiness to attend to aversive stimuli in a nonjudgmental and accepting attitude. By not being trauma focused, mindfulness is less aversive; by being trauma inclusive, it facilitates awareness and engagement with troubling aspects of PTSD.

In theory, mindfulness could be useful in the treatment of PTSD, in teaching patients to stay in the moment and not dwell on past or future events beyond their control. This less reactive mode of coping may provide a way for people with PTSD to feel a greater sense of control and be less avoidant, which may lead to improved quality of life and emotional well‐being (8, 22). Indeed, participation in MBSR has been associated with reduced intrusive ideation, worry, anxiety, and emotional distress and increased sense of control (17) and emotional well‐being (12, 23, 24). This reduced distress has been found to endure upon three‐month, six‐month, and four‐year follow up. Of the randomized controlled trials comparing MBSR to treatment as usual, 11 demonstrated improved mental health symptoms with overall medium effect sizes (i.e., MBSR had a clinically meaningful response compared with the control group) (25). MBSR has been shown to reduce symptoms of chronic pain, anxiety, depression, and PTSD (24, 26, 27).

In response to a growing consumer request for meditation to be offered to veterans with PTSD, in 2010 the Veterans Health Administration (VHA) prioritized a need for additional research on the effectiveness of meditation in veterans with PTSD. In this article, we present the results of a multisite randomized controlled trial on the use of MBSR for treatment of veterans with PTSD.

## Methods

### Study Design

From January 2012 to September 2013, U.S. military veterans diagnosed as having PTSD were randomly assigned to receive eight weeks of either MBSR or present‐centered group therapy (PCGT) at three clinical research sites in VA Medical Centers located in the southeastern United States. We hypothesized that MBSR would improve symptoms of PTSD over a nine‐week follow up compared with PCGT. The Clinician‐Administered PTSD Scale for DSM‐IV (CAPS‐IV) served as the primary outcome to evaluate treatment efficacy, and secondarily, to examine the treatment's effects on PTSD symptom clusters and rates of response. Outcome assessments were obtained at baseline and weeks 3, 6, and 9 (primary endpoint). As an exploratory measure of short‐term durability, these assessments were repeated posttreatment at week 16. All sites obtained local institutional review board approval prior to engaging in human subjects research. The study was monitored by an independent data monitoring committee. All participants received full explanation of the purpose, procedures, risks, benefits, and alternatives to treatment and provided informed consent and privacy authorization prior to study entry.

### Participants

Veterans of any combat or noncombat era were included if they were able to provide informed consent; were 19 to 65 years of age (inclusive); had a diagnosis of PTSD; had a CAPS‐IV score of ≥45 for the week prior to randomization; had no substance use disorders (except nicotine and/or caffeine) for two weeks prior to randomization; had no diagnosis of bipolar I disorder, schizophrenia, or a schizoaffective disorder; and were not actively considering suicide or homicide. Participants were excluded if they had current psychotic symptoms that in the investigator's opinion impaired their ability to provide informed consent or made participation unsafe, a severe cognitive disorder (e.g., dementia or severe traumatic brain injury), or a clinically significant unstable or severe medical condition that would contraindicate study participation or expose the participant to undue risk. Patients taking psychotropic medications were included if the medication had been taken for at least four weeks and if the dosage had been stable for two weeks prior to randomization and remained stable throughout the study (dose reductions due to unwanted side effects were allowed). Patients taking pain medication were included if their dosage had been stable for the two weeks prior to randomization. Patients were excluded if they would be receiving concurrent cognitive‐behavioral therapy, cognitive processing therapy, or prolonged exposure therapy during the study.

### Screening Procedures and Assessments

Baseline assessment included psychiatric evaluation; review of psychotropic medication and psychotherapy treatment history; recording of demographic data and disability status; inventory of general medical conditions; and review of recent physical examination and laboratory tests, including urine screen for drugs of abuse. A trained clinical research coordinator conducted the baseline Mini‐International Neuropsychiatric Interview, a structured clinician‐administered inventory that assessed current and lifetime *DSM‐IV* disorders (28). At baseline, the participants were asked whether they had a preference regarding treatment assignment (MBSR, PCGT, or none).

The CAPS‐IV (29) was used to confirm diagnosis of PTSD and to evaluate changes in PTSD symptoms. Trauma exposure was based on the participants’ verbal history, supplemented by the CAPS‐IV Life Events form, and included combat, noncombat, and/or sexual trauma events. Participants were instructed to focus on the worst incident of trauma, the one that had most likely resulted in the diagnosis of PTSD. CAPS‐IV assessments were rated for the past week and, if needed, the baseline CAPS‐IV was repeated so that the baseline score was collected the week prior to starting the intervention. The majority of the CAPS‐IV assessments were conducted by a trained independent assessor at each site who was blind to the treatment (single‐blind assessment). CAPS‐IV interviews were audio recorded and submitted to a CAPS‐rating fidelity monitor for review.

The PTSD Checklist—Self‐Report (PCL), a 17‐item self‐report scale, was used to evaluate PTSD symptoms. The Five Facet Mindfulness Questionnaire–Self‐Report (FFMQ) was used to assess five facets of mindful living (observing, describing, acting with awareness, nonjudging of inner experience, and nonreactivity to inner experience) (30, 31, 32). The nine‐item self‐report Patient Health Questionnaire was used to evaluate depression.

### Interventions

The MBSR groups met for eight weekly, 90‐minute sessions and a six‐hour retreat prior to week 6. MBSR training included the body scan meditation (a gradual moving of attention through the body from feet to head accompanied by awareness of breathing and other bodily sensations while lying in a supine position), sitting meditation (a focusing on the awareness of breathing, bodily sensations, thoughts, and emotions, practiced while sitting upright on a chair or cushion), and mindful stretching (exercises practiced with awareness of breathing and intended to develop mindful awareness during movement). Participants were given two guided meditation CDs to practice at home. In‐class didactic material emphasized the systematic development of mindful awareness and its application in everyday life. The six‐hour retreat included extended practice of the mindfulness body scan and mindful sitting, walking, stretching, and eating.

PCGT was selected as the comparison treatment because of its well‐established use as a control for the nonspecific effects of a group‐based intervention (33). The PCGT groups met for eight weekly, 90‐minute sessions and a lunch gathering prior to week 8. PCGT facilitated the expectation of symptom reduction, normalization of PTSD symptoms through education, decreased isolation, shared support, shared positive experiences with other veterans with similar symptoms, experience of an atmosphere of safety, and awareness and objectivity of how PTSD affects one's daily life. These groups had a present focus on current events that avoided discussion of traumas. PCGT treatment was psycho‐educational and included discussion of everyday problems of group members and of how PTSD created or intensified these problems. The PCGT participants were assigned to keep a journal and had a two‐hour lunch gathering to partially control for the MBSR retreat. The duration differences in the PCGT lunch and MBSR retreat reflect an uncontrolled difference in total treatment time. Both interventions provided an instruction manual to guide the therapists in conducting the treatment.

### Adherence and Attendance

To minimize attrition, the number of assessments was limited to decrease participant burden. The research coordinators called the participants to remind them of their appointments. Assessment visits were scheduled at convenient times for the participants and often were paired with other appointments. Participants were paid a small fee to offset out‐of‐pocket expenses for attending the assessment visits but not for the MBSR or PCGT sessions. In keeping with the intent‐to‐treat design, participants could remain in the study for assessments even if they dropped out of treatment.

### Fidelity Monitoring

To ensure consistent delivery of the MBSR and PCGT curricula, supervisors held separate monthly teleconferences with the MBSR therapists and with the PCGT therapists. Curriculum delivery was reviewed and potential threats to fidelity were discussed. Adherence to the MBSR and PCGT curricula was assessed by an independent fidelity monitor who listened to a random selection of 30% of the audio‐recorded sessions. The fidelity monitor rated the MBSR sessions using an adapted version of the Mindfulness‐Based Cognitive Therapy Adherence Appropriateness and Quality Scale (34, 35). The fidelity monitor used a similar scale to evaluate the PCGT group (replacing MBSR with PCGT techniques). If a therapist deviated from the treatment guide, the fidelity monitor provided the information to the MBSR or PCGT supervisors, who then worked with the therapist to either remediate or be replaced.

### Statistical Analyses

Using a blocking strategy and stratification based on site, we randomized the sample, using the Dallal software (Tufts University) in a 1:1 allocation. Analyses adhered to a modified intent‐to‐treat principle, classifying participants by randomized treatment condition and attendance in at least one group therapy session. To examine the balance across randomized treatment groups, we compared the two groups (MBSR vs. PCGT control) on baseline demographic and clinical characteristics using t tests for continuous variables, Wilcoxon tests for ordinal variables, and chi‐square tests for categorical variables. These analyses were used to identify potential confounding variables to be used as covariates in subsequent analyses. Only baseline variables that differed significantly between treatment groups and were correlated at 0.30 or higher with the outcome (CAPS‐IV scores) were included as covariates. Unless stated otherwise, each statistical test was conducted with a two‐tailed alpha of 0.05. We calculated medians, means, and standard deviations, and change from baseline for the CAPS‐IV (primary outcome) and all other scales by treatment condition over time. We calculated the within‐treatment condition rate of response, defined as a ≥30% decrease in CAPS‐IV score. Both between‐treatment condition and within‐treatment condition effect sizes were calculated: Cohen's d for continuous outcomes and the number needed to treat (NNT) for response rates. The effect size conveys a description of the magnitude of change that is independent of sample size (36). A 95% confidence interval accompanies each effect size to guide interpretation. For all analyses, week 9 was considered the end of the acute phase of treatment.

A three‐level mixed‐effects linear regression analysis was used to compare MBSR and PCGT treatment on total CAPS‐IV score during the trial. The data structure involved repeated measures over time nested within a participant, who in turn, was nested within a therapy group. Each model included up to four repeated assessments of the CAPS‐IV as the dependent variable (baseline and weeks 3, 6, and 9). The models included a random intercept, a random slope, and fixed effects for treatment condition, time, and the stratification variable (site). Because the interventions were in group formats, a random effect for each therapy group accounted for correlation of outcomes due to idiosyncratic group therapy factors. Likelihood ratio (LR) tests examined the incremental contribution of the treatment by time interaction. The decision rule called for rejection of the null hypothesis of no treatment effect if this interaction was statistically significant (two‐tailed α = 0.05). In addition, LR tests compared model fit that included a treatment‐by‐site interaction and a first‐order autoregressive covariance structure (37).

The secondary outcomes (mindfulness, depression, PTSD symptom clusters, and PTSD response rates) were analyzed in separate three‐level mixed‐effects linear regression models by using the strategy described above for the primary outcome. The categorical outcome measure (response status ≥30% decrease in CAPS‐IV score from baseline) was examined with mixed‐effects logistic regression analysis. The Hochberg multiplicity adjustment (38) was used for analyses of all secondary outcomes, with a familywise alpha level of 0.05.

## Results

### Participants

The CONSORT diagram (online supplement) shows the number of participants who provided informed consent (N=254), were randomly assigned to a study condition (N=214), included in the analysis (N=191), completed the week 9 assessment visit (N=142), and completed the week 16 assessment visit (N=130). Reasons for not being randomly assigned (16%) included having a subthreshold CAPS‐IV score (N=15), alcohol or drug use disorder (N=4), bipolar I disorder (N=5), not returning for random assignment (N=6), withdrawing or moving away (N=7), or other (N=3). Reasons for not being included in the analysis (N=23) were being lost to follow‐up (N=5), withdrawing consent (N=4), or entering addiction rehabilitation treatment (N=2) in the MBSR group and being lost to follow‐up (N=7), withdrawing consent (N=3), and relocating (N=2) in the PCGT group.

In the MBSR group, 71 remained in the study; however, only 65 (68%) attended the week 9 assessment. Reasons for not completing the week 9 assessment visit (32%) in the MBSR group included being lost to follow‐up (N=14), withdrawing (N=5), moving (N=2), investigator withdrawing (N=1), noncompliance (N=2), and other (N=1). Due to absence of a blinded rater, the CAPS‐IV assessment was not completed for three participants who attended the week 9 assessment, leaving 62 in the CAPS‐IV primary end‐point analysis. Six participants missed week 9 but continued in the study and attended the week 16 assessment.

In the PCGT group, 81 remained in the study; however, only 77 (81%) attended the week 9 assessment. Reasons for not completing the week 9 assessment visit (19%) in the PCGT group included being lost to follow‐up (N=8), investigator judgment that it was in the participant's best interest to exit (N=2), work (N=2), unrelated adverse event (N=1), and being detained in jail (N=1). Four participants missed week 9 but continued in the study and attended the week 16 assessment.

Reasons for not completing the week 16 assessment (36%) in the MBSR group included being lost to follow‐up (N=7) and moving (N=3). Due to unavailability of a blinded rater, one CAPS‐IV assessment was missing. Reasons for not completing the week 16 assessment (27%) in the PCGT group included being lost to follow‐up (N=6), moving (N=4), and unknown (N=2). Due to unavailability of a blinded rater, one CAPS‐IV assessment was missing.

### Baseline Demographic and Clinical Characteristics

No significant differences were observed between groups in baseline demographic and clinical characteristics (Table 1). On average, the sample was 84% male, one‐third Caucasian, and two‐thirds African American or another racial‐ethnic minority. Average age was 51, with a broad distribution of marital status. Fifty‐six percent had served in the Army, 16% had served in the military post‐9/11, 48% had experienced combat‐related trauma, 76% had more than a high school education, and 80% were currently not working.

**Table 1:** Baseline demographic and clinical characteristics of veterans with posttraumatic stress disorder (PTSD) included in the analysis^a^

	**MBSR (N=96)**		**PCGT (N=95)**		
**Characteristic**	**N**	**%**	**N**	**%**	**p**
Sex					.87
Male	80	83.3	80	84.2	
Female	16	16.7	15	15.8	
Race					.95
White	30	31.3	31	33.3	
Black	63	65.6	59	63.4	
Other/unknown	3	3.1	5	5.2	
Education					.71
Less than college	22	22.9	24	25.3	
Some technical school or college	36	37.5	37	39.4	
Technical school/associate degree	10	10.4	14	14.9	
College degree or higher	28	29.2	19	20.0	
Marital status					.58
Single	13	8.3	17	17.9	
Married	43	44.8	35	36.8	
Separated	14	14.6	8	8.4	
Divorced	25	26.0	32	33.7	
Widowed	1	1.0	1	1.0	
Employment					.06
Unemployed	28	21.5	27	28.4	
Employed	22	22.9	17	17.9	
Retired	9	9.7	11	11.6	
Disabled	32	34.4	41	43.6	
Receiving SSDI/SSI income	22	22.9	28	29.5	.44
Branch of military service					.61
Army	52	54.2	55	58.5	
Navy	12	12.5	9	9.6	
Marines	14	14.6	9	9.6	
Air Force	4	4.2	8	8.5	
National Guard/Coast Guard	3	3.1	5	5.3	
Combination	11	11.5	8	8.5	
Period of service					.76
Korea	1	1.0	0	.0	
Vietnam	39	40.6	35	36.8	
Gulf War (I)	18	18.8	25	26.6	
Gulf War (II), after 9/11	16	16.7	14	14.9	
Other	22	22.9	20	21.3	
Type of trauma					.70
Combat	51	54.8	48	51.1	
Military, noncombat	23	24.7	29	30.9	
Sexual	10	10.8	11	11.7	
Civilian	9	9.7	6	6.4	
Concurrent DSM‐IV disorders					
Major depression	54	56.3	53	55.8	.95
Dysthymia	9	9.4	7	7.4	.62
Panic	33	34.4	27	28.4	.38
Agoraphobia	46	47.9	38	40.0	.27
Social phobia	17	17.7	24	25.3	.20
Obsessive‐compulsive	8	8.3	5	5.3	.40
Alcohol dependence (past 12 months)	13	13.5	12	12.6	.85
Alcohol abuse (past 12 months)	3	3.1	6	6.3	.30
Substance dependence (past 12 months)	9	9.4	9	9.5	.98
Substance abuse (past 12 months)	1	1.0	2	2.1	.55
Eating disorders	6	6.3	5	5.3	.77
	M	SD	M	SD	p
Age (years)	51.7	10.9	51.0	11.4	.67
Service‐connected disability (%)	34.0	35.2	35.8	35.4	.73
Medical service connection (%)	14.3	23.2	16.6	24.9	.52
Psychiatric service connection (%)	2.9	12.1	3.6	16.5	.77
PTSD service connection (%)	15.7	28.1	18.1	29.6	.58
Duration of military service	7.4	6.9	8.6	7.8	.29

a MBSR, mindfulness‐based stress reduction; PCGT, present‐centered group therapy; SSDI/SSI, Social Security Disability Insurance/Supplemental Security Income.

### Adherence to Group Therapy Sessions and Outcomes Assessments

Of participants who attended at least one group therapy session (analyzed sample), fewer MBSR participants than PCGT participants completed the outcomes assessments (68% vs. 81%, respectively, at week 9; 64% vs. 73%, respectively, at week 16; online supplement), but the difference did not reach statistical significance. Table 2 shows the distribution of attendance at each group therapy session. Although the MBSR group had more participants attending fewer group sessions compared with the PCGT group, no significant differences were observed in the number of group sessions attended between treatment arms (p=0.905). The participants’ adherence to treatment (attendance) did not correlate with CAPS‐IV or FFMQ outcomes at week 9 or 16.

**Table 2:** Frequency counts of veterans attending group sessions and correlation of outcome with percentage of PTSD group sessions attended^a^

**Number of Groups Attended**	**MBSR (N)**	**PCGT (N)**	**Correlation with % attendance**	**P**
0	11	12		
1	8	2		
2	4	3		
3	7	1		
4	7	7		
5	7	8		
6	8	18		
7	12	29		
8	18	27		
				
CAPS‐IV endpoint				
Week 9			.10	.22
Week 16			.07	.41
FFMQ endpoint				
Week 9			−.08	.37
Week 16			−.08	.35

a CAPS‐IV, Clinician‐Administered PTSD Scale for DSM‐IV; FFMQ, Five Facet Mindfulness Questionnaire; MBSR, mindfulness‐based stress reduction; PCGT, present‐centered group therapy.

### Outcome Measures

No statistically significant differences were observed between the MBSR and PCGT groups in terms of the primary (CAPS‐IV) or secondary outcomes, except for the PCL (Tables 3 and 4). A statistically greater improvement was observed in PTSD based on the self‐reported PCL in the MBSR group compared with the PCGT group (Table 4). The participants’ baseline treatment preference (MBSR vs. PCGT vs. none) had no moderating effect on CAPS‐IV scores at week 9 (p=0.734) or 16 (p=0.741). Rates of response, defined as CAPS‐IV reduction ≥30%, did not statistically differ between groups (45.2% MBSR vs. 37.7% PCGT, p=0.293, between‐group NNT=12). Rates of remission, defined as CAPS‐IV score ≤45, did not statistically differ between groups (30.7% MBSR vs. 27.3% PCGT, p=0.662, between‐group NNT=30).

**Table 3:** Change from baseline for primary and secondary outcomes for veterans assigned to mindfulness‐based stress reduction (MBSR) or to present‐centered group therapy (PCGT)^a^

	**Week 3**		**Week 6**				**Between‐group differences**			**Week 16**		
**Scale**	**M**	**SD**	**M**	**SD**	**p** ^b^	**d**	**M**	**SD**	**95% CI**	**M**	**SD**	**p** ^b^
CAPS‐IV (total score)					.53	−.26	−6.4	24.8	−14.78, 1.97			.97
MBSR	−12.9	19.9	−13.6	24.8						−18.3	30.6	
PCGT	−9.3	17.9	−17.3	21.2						−18.2	25.1	
CAPS‐B (re‐experiencing)					.81	−.16	−1.5	9.6	−4.75, 1.71			.92
MBSR	−4.2	8.4	−3.6	8.8						−6.2	10.9	
PCGT	−4.6	7.6	−6.6	8.1						−6.4	9.2	
CAPS‐C (avoidance and emotional numbing)					.42	.17	−2.0	11.6	−5.90, 1.95			.98
MBSR	−5.2	9.2	−6.1	11.7						−7.5	18.9	
PCGT	−3.1	9.3	−7.1	10.8						−7.5	11.3	
CAPS‐D (hyperarousal)					.35	−.36	−2.9	8.4	−5.74,. 08			.85
MBSR	−8.5	7.3	−3.9	8.8						−4.6	9.1	
PCGT	−1.6	7.1	−3.5	6.9						−4.3	8.8	
PTSD Checklist (self‐report)					.57	−.14	−1.1	13.8	−6.71, 2.9			.68
MBSR	−4.3	12.0	−6.3	13.3						−6.6	14.4	
PCGT	−2.8	9.5	−6.7	11.5						−5.5	15.7	
FFMQ (self‐report)					.40	.21	3.1	14.8	−1.86, 8.11			.25
MBSR	1.9	12.2	3.9	14.6						6.3	20.5	
PCGT	−1.0	10.6	.5	9.9						2.6	14.9	
PHQ‐9 (self‐report)					.80	−.25	−1.5	6.5	−3.71, 0.64			.81
MBSR	−1.1	5.5	−2.0	5.7						−3.0	7.4	
PCGT	−.9	4.7	−2.5	5.9						−2.6	8.0	

a CAPS‐IV, Clinician‐Administered PTSD Scale for DSM‐IV (total score range 0–136, CAPS‐B range 0–40, CAPS‐C range 0–56, CAPS‐D range 0–40; higher score indicates more severe PTSD symptoms); FFMQ, Five Facet Mindfulness Questionnaire (range 39–195; higher score indicates greater mindfulness); PHQ‐9, nine‐item Patient Health Questionnaire (range 0–27; higher score indicates more severe depression).

b p values are from the mixed model procedure.

**Table 4:** Primary and secondary outcomes for veterans assigned to mindfulness‐based stress reduction (MBSR) or to present‐centered group therapy (PCGT)**^a^**

	**Baseline**		**Week 3**		**Week 6**		**Week 9**			**Week 16**	
**Scale**	**M**	**SD**	**M**	**SD**	**M**	**SD**	**M**	**SD**	**p** ^b^	**M**	**SD**
CAPS‐IV (total score)									.58		
MBSR	84.3	19.5	71.1	24.3	68.6	26.9	59.1	27.1		64.8	30.8
PCGT	80.7	16.7	70.4	23.9	63.1	23.9	64.5	28.2		62.9	28.3
CAPS‐B (re‐experiencing)									.73		
MBSR	23.4	8.2	18.9	9.7	19.0	9.4	14.8	10.3		17.0	10.8
PCGT	23.0	6.9	18.3	8.9	16.1	8.9	16.5	9.8		16.8	9.9
CAPS‐C (avoidance and emotional numbing)									.42		
MBSR	34.6	8.4	29.2	11.4	27.9	12.3	24.9	12.5		26.5	13.8
PCGT	32.4	9.7	28.7	10.9	25.2	13.9	25.7	13.8		25.3	13.6
CAPS‐D (hyperarousal)									.49		
MBSR	26.3	6.3	22.9	6.6	21.7	8.3	19.5	7.8		21.3	8.9
PCGT	25.2	5.4	23.4	8.2	21.8	7.2	22.3	7.9		20.8	8.5
PTSD Checklist (self‐report)									.04		
MBSR	63.1	12.2	58.7	13.9	56.7	15.8	53.8	17.4		56.2	16.5
PCGT	58.7	12.2	55.3	12.4	57.9	14.2	52.6	14.4		53.8	15.9
FFMQ (self‐report)									.48		
MBSR	109.9	21.0	110.9	20.1	114.7	20.6	116.9	20.2		117.2	23.6
PCGT	112.1	16.2	111.0	15.7	113.6	15.1	114.6	16.8		116.1	16.7
PHQ‐9 (self‐report)									.54		
MBSR	27.7	6.7	26.7	6.3	25.0	6.5	23.8	7.6		24.0	7.6
PCGT	26.8	6.9	25.6	6.2	24.0	6.1	24.5	7.4		24.5	7.4

a CAPS‐IV, Clinician‐Administered PTSD Scale for DSM‐IV (total score range 0–136, CAPS‐B range 0–40, CAPS‐C range 0–56, CAPS‐D range 0–40; higher score indicates more severe PTSD symptoms); FFMQ, Five Facet Mindfulness Questionnaire (range 39–195; higher score indicates greater mindfulness); PHQ‐9, nine‐item Patient Health Questionnaire (range 0–27; higher score indicates more severe depression).

b p values are from the mixed model procedure.

Statistically significant correlations were observed between reduction in CAPS‐IV scores and improvement in FFMQ scores in each group (MBSR, –0.509, p=0.01; PCGT, –0.337, p=0.001). To check the comparability of the direction and intensity of the combined CAPS‐IV and FFMQ result between the two groups, the difference in the CAPS‐IV and FFMQ scores within each treatment group was calculated and the correlation of the difference was then determined to be 0.927 (representing the correlation of the direction and intensity of the combined CAPS‐IV and FFMQ results between the two groups, which was high and statistically significant, p=0.023). These results show that the relationship between the CAPS‐IV and FFMQ was comparable between the two groups.

### Adverse Events

Both treatments were well tolerated. In the MBSR group, five unrelated serious adverse events occurred: one psychiatric inpatient admission for suicidal ideation of a participant who was randomly assigned to MBSR but had not yet attended a treatment session, one participant with a wisdom tooth infection that resulted in medical admission, and three participants with psychiatric inpatient admissions for suicidal ideation. In the PCGT group, two unrelated serious adverse events occurred: one participant with a psychiatric inpatient admission for suicidal ideation and one participant with medical hospitalization for hypotension.

## Discussion

In this multisite randomized controlled study of veterans diagnosed as having PTSD, the MBSR and PCGT interventions improved PTSD symptoms over time, with no significant differences between groups on the CAPS‐IV scale. Although the results of the self‐reported PTSD Checklist differed significantly between groups at week 9, this difference was not maintained at week 16. Although PCGT was selected as a control group, it has many active therapeutic elements, including group cohesion, validation from other veterans, health education, and psychosocial support, and these attributes may explain why the two treatment groups yielded similar results. For example, PCGT was shown to have similar positive outcomes as a group‐based exposure therapy in a VA multisite study of male Vietnam veterans with PTSD (33). A larger sample size may be needed to better differentiate responses between the two treatments.

It is not clear why a significant difference between treatments was found on the self‐reported PCL versus the CAPS‐IV. Perhaps veterans in the MBSR group felt greater efficacy and control, which led to a perception of clinical improvement that was not noted by investigators charting symptoms. Mindfulness is an approach that may improve appraisals of secondary control (39) and acceptance of thoughts and emotions (40).

Strengths of our study include its multisite design, active control group (i.e., PCGT rather than waitlist control), blinded primary outcome ratings, fidelity monitoring, large minority representation, and randomized approach. This study was limited by its high attrition rate and brief MBSR group sessions. We shortened MBSR from its usual 160‐ or 180‐minute format to match the 90‐minute duration of the PCGT groups. Other researchers may want to include a psychoeducation overview prior to participants committing to an eight‐week MBSR treatment as a means to reduce attrition and may want to provide access to MBSR practice groups during long‐term follow‐up, as would be provided in real‐world settings.

A similarly designed study by Polusny et al. (26) found a greater decrease on the self‐reported PCL for the MBSR treatment group compared with the PCGT group, as well as significant improvements in CAPS‐IV and quality‐of‐life scores at week 17. Our study differed from the Polusny et al. study in several ways, including sample size, number of sites and therapists, demographic characteristics of the study sample, length of the MBSR group sessions, response to PCGT, primary outcome measure, and attrition rates. These factors may explain the differences in the findings. Our study enrolled more participants (N=214 vs. N=116) who scored approximately 15 points higher on baseline CAPS‐IV, and we used three sites instead of one. The multisite approach may have increased variability in patient selection, treatment delivery, and CAPS‐IV scores. Our study had substantial minority representation: two‐thirds were African American compared with only 8% in the Polusny et al. study. To balance the treatment interventions, we limited the MBSR groups to 90‐minute sessions to match the recommended PCGT session time, whereas Polusny and colleagues held MBSR groups for 2.5 hours and PCGT groups for 90 minutes, thus providing more experiential practice and reinforcement for the MBSR groups. Compared with Polusny et al., our study had higher attrition rates overall, most notably in the MBSR group (10% vs. 32%, respectively).

Our results were included in a meta‐analysis (41) of nine randomized controlled trials of PTSD, which found an overall effect size of –0.34 (p<0.001, 95% CI=–0.48, –0.18) for mindfulness‐based meditation. All but one of these trials had an active control group. In addition to these studies, a pilot study conducted to examine the effects of MBSR versus PCGT among veterans with PTSD found that MBSR was associated with changes in functional response to exposure to the stressor of Iraq combat‐related slides and sounds in the anterior cingulate, parietal cortex, and insula (27). This small comparison study showed a reduction in both CAPS‐IV and FFMQ scores in the MBSR group but not in the PCGT group, with the effects maintained at six months.

## Conclusions

In conclusion, MBSR did not have a significant advantage over PCGT in our sample. The overall small effect sizes of mindfulness‐based meditation should be viewed with caution in the context of larger effect sizes of trauma‐focused behavioral psychotherapies. As with all complementary and integrative health approaches, mindfulness‐based meditation should be a supplement to, not a replacement for, trauma‐focused behavioral psychotherapies. Additional studies are needed to better understand the effects of MBSR for the treatment of PTSD.

## Supporting information

Supplementary Material
