# Supplementary figures and images for: A Multisite Randomized Controlled Trial of Mindfulness‐Based Stress Reduction in the Treatment of Posttraumatic Stress Disorder
Source: Psychiatr Res Clin Pract. 2018 Sep 13;1(2):39–48. doi: 10.1176/appi.prcp.20180002 (PMC8189576; doi:10.1176/appi.prcp.20180002)

**CONSORT Flow Diagram**  
**Randomized Controlled Trial of MBSR vs PCGT in Veterans with PTSD**

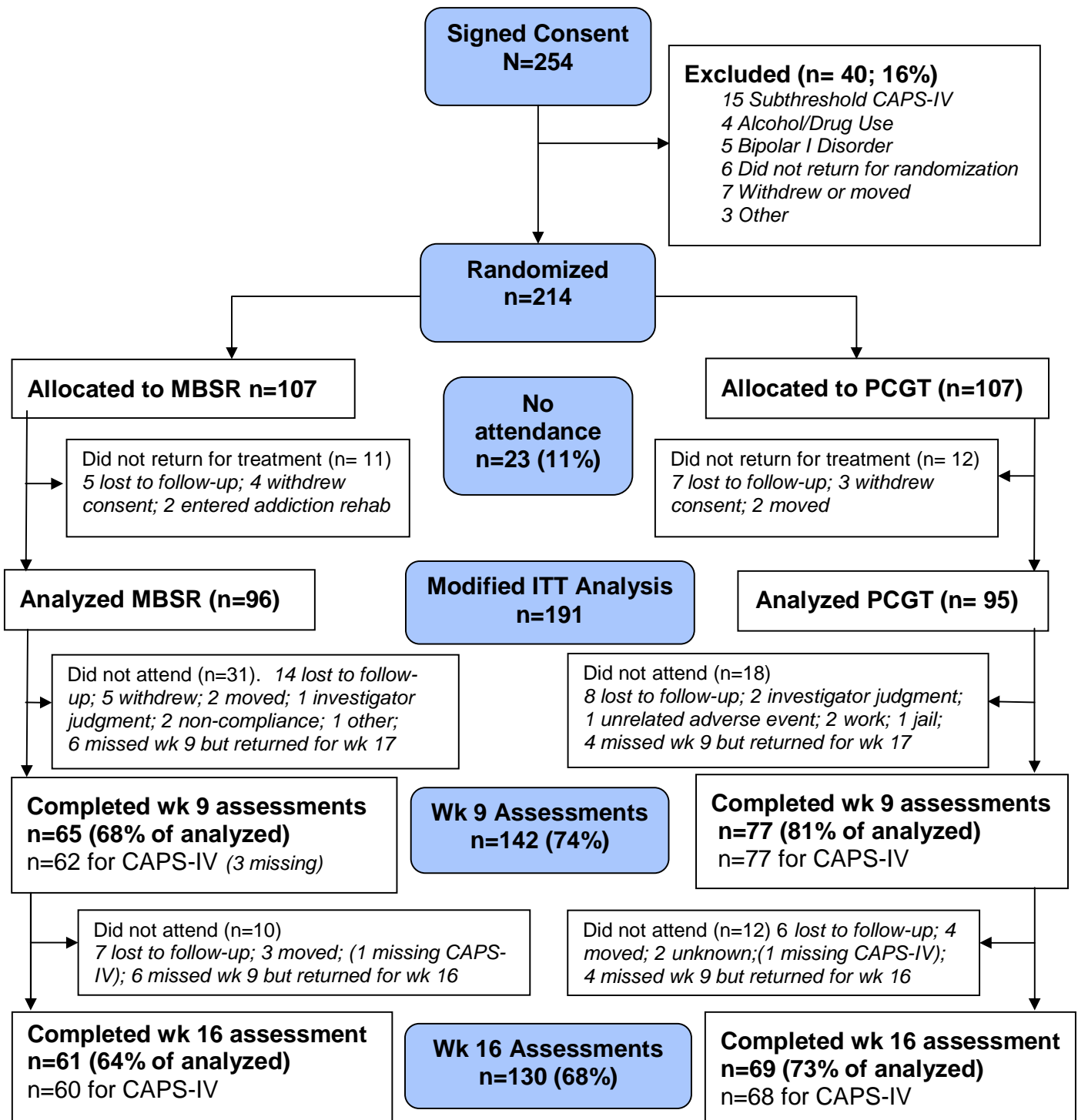

Supplement: Supplementary file 1 — Supplementary Material [file RCP2-1-39-s001.pdf]
